# Lesbian and bisexual women's experiences of aversion therapy in England

**DOI:** 10.1177/09526951211059422

**Published:** 2022-01-06

**Authors:** Helen Spandler, Sarah Carr

**Affiliations:** 6723 University of Central Lancashire, UK; 1724 Independent Researcher, UK

**Keywords:** behaviour therapy, female, homosexuality, psychiatry, psychology

## Abstract

This article presents the findings of a study about the history of aversion therapy as a treatment technique in the English mental health system to convert lesbians and bisexual women into heterosexual women. We explored published psychiatric and psychological literature, as well as lesbian, gay, and bisexual archives and anthologies. We identified 10 examples of young women receiving aversion therapy in England in the 1960s and 1970s. We situate our discussion within the context of post-war British and transnational medical history. As a contribution to a significantly under-researched area, this article adds to a broader transnational history of the psychological treatment of marginalised sexualities and genders. As a consequence, it also contributes to LGBTQIA+ history, the history of medicine, and psychiatric survivor history. We also reflect on the ethical implications of the research for current mental health practice.

## Introduction

This article is an output from our research project about the ‘treatment’ of female homosexuality in the English mental health system in the post-war period, when homosexuality was classified as a mental disorder (Carr and Spandler, 2019; Spandler and Carr, 2020, 2021). Whilst there is some relevant research about the broader history of psychiatry, psychology, and homosexuality, especially in the US (e.g. Bayer, 1987; Herman, 1995); aversion therapy (e.g. Davison, 2020a; Dickinson, 2015), and lesbians' experience of psychiatry (e.g. Jennings, 2008; Oram and Turnbull, 2001), this article focuses specifically on the practice of aversion therapy to reorientate women's sexuality. Although there is some research documenting gay men's experiences of being subjected to this treatment, little is known about women's experiences. Our research focused on mental health settings within the National Health Service (NHS) so did not include conversion therapies practised within religious or other settings.

## Mental health services, homosexuality, and aversion therapy

Campaigning efforts to declassify homosexuality as a mental disorder have been relatively well documented, especially in the US context (Bayer, 1987; Drescher, 2015; Drescher and Merlino, 2007; Minton, 2002). Its pathologisation is now widely discredited within the psychiatric profession and allied ‘psy’ disciplines (including psychology, psychotherapy, and other allied disciplines). For example, in 2017 the president of the British Royal College of Psychiatrists issued a welcome, if overdue, apology for psychiatry's complicity in the oppression of gay people (Strudwick, 2017). In the post-war period, the psy professions experimented with a range of ways to try and reorientate people's sexuality. Aversion therapy was probably the best known and most controversial.

Aversion therapy was practised in recognised mental health institutions by psychiatrists and psychologists from the 1950s to the 1980s. It was one of a range of behavioural modification techniques underpinned by behaviourism, arguably one of the most ‘successful’ orientations in British clinical psychology, at least in terms of funding, longevity, and institutional support (Marks, 2015). Aversion therapy was used to treat numerous conditions, including compulsions, obsessions, alcoholism, gambling, and enuresis (bed-wetting), as well as ‘paraphilic’ behaviours, including frigidity, exhibitionism, fetishism, voyeurism, transvestism, and homosexuality (James, 1962). In this practice, underpinned by a crude behaviourist psychology, homosexuality was viewed as faulty learned behaviour that could be unlearned. In the procedure, homosexual patients were exposed to sexualised images of individuals of the same sex, whilst simultaneously being subjected to either electric shocks or emetic drugs to make them vomit. This was intended to ‘condition’ the patient to associate the sexual stimulus with unpleasant sensations in order to stop the targeted behaviour (homosexuality) and promote heterosexuality through positive reinforcement (the cessation of pain).

The pathologisation and treatment of homosexuality is a common feature of queer and LGBTQIA+  histories.^1^ For example, Tommy Dickinson's *Curing Queers* (2015) explored the experiences of ‘homosexual’ and ‘transvestite’ men and the nursing staff who administered aversion therapy in Britain. Yet despite this, the behaviourist therapeutic paradigm remains ‘almost entirely unmapped’, and there has been, up to very recently, ‘an astonishing lack of historical scholarship on the history of aversion therapy’ (Davison, 2020a: 91). For example, Rutherford (2006, 2009) explored the history and ethics of applied behavioural analysis in the US context, but did not include aversion therapy for sexual orientation.

According to Davison (2020a), the use of aversion therapy to treat homosexuality spanned a period of about 60 years, comprising three distinct ‘waves’ during the middle of the 20th century. A few recorded cases in the US during the 1920s and 1930s (Max, 1935) were followed by extensive research trials in Czechoslovakia in the 1950s, known as the ‘Prague experiments’. British behaviourists propelled a more sustained and enthusiastic ‘third wave’, from the late 1950s through the 1960s, in England as well as other English-speaking countries, such as Australia, New Zealand, and the US (Davison, 2020a, 2020b). The treatment was gradually phased out after 1973, when key psychiatric organisations began to remove homosexuality from their categories of ‘mental disorders’. However, it was not until 2016 that the World Psychiatric Association finally declared that any form of conversive, ‘reparative’, or reorientation therapy was unethical, unscientific, and harmful (Strudwick, 2017).

## Aversion therapy and behaviourism in Britain

Although British behavioural psychologists did not ‘invent’ aversion therapy, it has been suggested that they were the most positive about its efficacy in treating homosexuality. For example, Davison (2020a) argues that some British behaviourists ignored, or at least downplayed, results from the extensive ‘Prague experiments’, which were distinctly cautious about the use and effectiveness of using aversion therapy to treat homosexuality. For example, ‘an entire body of clinical research conducted in Czechoslovakia in the 1950s went mostly unacknowledged in the West and, when cited, was artfully cherry-picked for the most favourable gloss’ (ibid.: 91).

It needs to be borne in mind that behaviourism was on the ascendancy at the time, and was fiercely competing with psychoanalysis to become the dominant psychological approach to treating mental disorders. Behavioural modification techniques were promoted as cheaper, quicker, and more effective than prolonged psychotherapy, and this was important in the context of the budget constraints of a post-war nationalised mental health service (Marks, 2015). Behaviour therapists made ‘considerable use of various aversive techniques for homosexuals, much more so than any other behavioural approach’ (Davison and Wilson, 1973: 693). Hans Eysenck, one of the leading proponents of behaviourism, who infamously described aversion theory as ‘just like a visit to the dentist’ (Tatchell, 1972), helped establish the King’s College Institute of Psychiatry at the Maudsley Hospital in London as a key site for the development of aversion therapy in England and an institutional bedrock for the creation of transnational ‘networks of expertise’ (Davison, 2020a). Aversion therapy was used to treat homosexuality in many NHS psychiatric hospitals in England as well as the Maudsley, including Glenside (Bristol), Upton (Chester), Graylingwell (Chichester), Banstead (Surrey), and Crumpsall (North Manchester). Moreover, it appears British behaviourists may have ‘exported’ aversion therapy to the USA, Australia, and New Zealand. This was aided by the emigration of several British psychiatrists associated with behaviourism (Cramond, 1981), with British ‘transnational vectors of influence’ helping to spread its use (Brickell and Bennett, 2018).

Several clinicians and researchers in Britain were enthusiastic about its ‘exceptionally promising potential to eliminate homosexuality’ (Barlow, 1972: 480) and its potential to develop effective treatment programmes for other ‘sexual deviations’ (Rachman and Teasdale, 1969). Whilst there were critiques of aversion therapy within the profession, these tended to focus on the technicalities of treatments (e.g. Feldman and MacCulloch, 1972; Tomi and MacDonough, 1972), rather than the ethics of enforcing heterosexuality on individuals (see Whitlock, 1964, for a notable exception). Moreover, whilst the treatment was increasingly opposed by the emerging lesbian and gay movement, the behaviourists who pioneered these treatments received considerable accolades for their efforts within the discipline (Sansweet, 1975). For example, Feldman and McCulloch’s (1971) *Homosexual Behaviour: Therapy and Assessment* was described as ‘probably the most important [work] yet written on homosexuality’ and the authors praised for ‘displaying ingenuity and perseverance in their pioneering attempts at rigorous research in a difficult clinical area’ (Barlow, 1972: 479).

Notwithstanding all this, it is important to bear in mind that clinicians who actively ‘treated’ homosexuality, including behaviour therapists, were probably in the minority. For example, in a survey of British behavioural psychologists conducted in the early 1970s, the overwhelming majority said they would be more likely to try and help homosexuals ‘to be more comfortable with their sexuality’ and would not attempt coercive therapy (Davison and Wilson, 1973). However, worryingly, 13% said they would not treat a homosexual to help them be more comfortable with their sexuality and had ‘treated or would consider treating a homosexual so as to change him/her in a heterosexual direction when he/she did not want to change’ (ibid.: 690).

It also needs to be borne in mind that male homosexual behaviour was criminalised in the UK at the time. In this context, aversion therapy was offered to some men who had been charged with homosexuality activity, as a ‘softer’ option than going to prison. It has been suggested that it may have been more privileged, educated, and middle-class men who were offered aversion therapy, whilst the majority of working-class men arrested for engaging in same-sex relations were more likely to end up in prison, without the offer of ‘treatment’ as an alternative (Waters, 2017). Not surprisingly, however, these treatments were experienced as ineffective at best, punitive and damaging at worst, and resulted in lifelong negative effects on the men concerned (Dickinson, 2015; Stapleton, 1975).

Whilst female homosexuality was also classified as a mental disorder (‘sexual deviation’), same-sex-attracted women were less likely to receive aversion therapy. Unlike male homosexuality, women's homosexual behaviour was not directly criminalised in England and therefore tended to be hidden, socially regulated, and pathologised in less visible and documented ways (Oram and Turnbull, 2001). Therefore, these women were never faced with the Hobson's choice of being offered aversion theory instead of prison, and there is less known about their experiences.

British researchers have been aware that some women were subjected to these treatments, from accounts ‘scattered in the written and recorded testimonies of LGBT people’ (Dickinson *et al.*, 2012: 1347) and earlier studies (e.g. MacColloch and Feldman, 1967), but these have not been brought together or explored in any detail. Indeed, when we started this research, a fellow scholar in the field said it would be like ‘finding a needle in a haystack’ (Spandler and Carr, 2020).

## Challenges in researching women's experiences of aversion therapy

There are numerous challenges in conducting research about sexualities and health (Kneale *et al.*, 2019). This section focuses on some of the difficulties in trying to surface women's experiences in our research. First, most of the relevant psychiatric and psychological research literature published during the period in question assumed that *homosexual* and *homosexuality* referred to men. Whilst these terms were very occasionally prefixed by *female* when applied to women, this was not the case for men. *Lesbian* was rarely, if ever, used in the medical literature. This term was often preferred by women to highlight the female centred-ness of their desire and to promote a less medicalised view of their sexuality than *homosexuality*. Moreover, when articles did refer to women, or when women were included in research studies, their ‘data’ was frequently subsumed within the overall data set (e.g. Feldman and MacCulloch, 1964; MacCulloch and Feldman, 1967).

More recent research has documented this history from a lesbian, gay, and bisexual perspective, but this has also, understandably, foregrounded gay men's experiences of being subjected to these treatments (Dickinson, 2015; Dickinson *et al.*, 2012; Smith, Bartlett, and King, 2004). For example, Smith, Bartlett, and King (2004) interviewed 29 former patients who were treated for homosexuality in the UK. They managed to recruit two female patients, and interviewed one of them (the other dropped out because of personal commitments). However, the authors did not highlight any specific details relating to the woman's interview. This makes it very difficult to draw out any relevant information about women's specific experiences. This mirrors a broader problem in the clinical context, where mental health professionals assume that female homosexuality is just the mirror image of male homosexuality, with little awareness that women may have specific experiences (Spandler and Carr, 2020).

In addition, with increasing awareness of gender, as well as sexual, diversity (especially trans, intersex, and non-binary genders), identifying specific genders in historical research can be complex. Whilst research has indicated that some ‘transvestite’ men were treated with aversion therapy for ‘cross dressing behaviour’ (Dickinson, 2015), we did not find any obvious examples of women being subjected to aversion therapy for these reasons.^2^ Having said that, at least some of the people recorded as being treated as (male) transvestites may actually have been (trans) women (or, at least, might have been if the wider culture had enabled this). Indeed, after we completed our archive research, we found examples of trans women being treated with aversion therapy in an attempt to treat their (trans) gender orientation (e.g. Evans, 2019).

This serves as an important reminder that the definitions, diagnoses, and designations used in archival sources are not necessarily as clear-cut as they may first appear. For example, people treated for either gender or sexual reorientation may have had different, more complex, and less binary understandings of their own gender and sexuality than the language immediately available to them at the time (i.e. homosexual or heterosexual; male or female). Without the testimony of survivors, this is impossible to disentangle. Whilst we fully acknowledge that the histories of gender and sexuality are deeply entangled and evolving, we focused in our research on attempts to change women's *sexuality*.

## Our research methods

First, we identified and carefully read published British literature about aversion therapy in relation to homosexuality, as practised on British soil, looking for any references to women. We consulted all the major medical, psychiatric, and psychology journals (such as the *British Medical Journal*, the *British Journal of Psychiatry*, and the *British Journal of Psychology*) and psychology and psychiatry textbooks on treating ‘sexual deviations’, specialist journals and textbooks on behavioural psychology, and literature specifically about aversion therapy and homosexuality. Then we conducted a qualitative ‘bottom-up’ study of LGBT+ archives, published anthologies, and oral history collections to try and identify any women who referred to being subjected to psychiatric treatment.^3^ Where possible, we used various keyword searches such as ‘psychiatry/psychiatric’, ‘psychology/psychological’, ‘treatment’, ‘aversion therapy’, and so on. However, as most of the archives were not catalogued or digitised, and sometimes the text was not yet available to consult, this was often not possible. This, coupled with the fact that archives are always being added to, meant that the search was not exhaustive, and there is scope for more research at some future time.

We scanned all the material available in the archives listed, reading in detail anything that looked like it might have any relevant information for our research. As our focus was on treatments administered within the mental health system, we excluded examples of religious-based ‘conversion therapies’. The co-researcher and co-author of this study is an established mental health service user who experienced involuntary mental health treatments, including psychotherapy and hypnotherapy, to try and reorientate her sexuality between the ages of 18 and 19 (Carr, 2004). This experience was used to inform our research.

## Our research findings

We identified several examples of lesbians and bisexual women being subjected to a range of psychiatric treatments in England, including a few isolated examples of ECT, LSD, and various forms of psychotherapy (see Carr and Spandler, 2019). However, it was not always possible to establish from the archival data whether these treatments were administered specifically to ‘treat’ their sexuality or for other mental health-related reasons. With aversion therapy, this link was much clearer, as it was a recognised behavioural treatment for homosexuality at that time. Across all sources consulted, we found approximately 10 examples of women being treated with aversion therapy to treat their ‘homosexual behaviour’ in England (we did not find any examples in Wales, Scotland, or Northern Ireland). We say ‘approximately’ because, as we explain, we cannot be certain that some of the examples given are not duplicates. Most of the examples were from the early to mid 1960s, when the women were usually in their late teens or early 20s. As aversion therapy is rooted in behavioural psychology, it would usually have been administered by a psychologist, or sometimes a psychiatric nurse, usually with the endorsement of the medical director of the psychiatric hospital, a consultant psychiatrist.

It is important to note that we found several positive accounts of women's experiences of psychiatry, psychology, and psychotherapy (see also Jennings’ [2008] account of lesbians' encounters with post-war British psychiatry). In these cases, women often sought help for their sexuality, or were pressurised into seeking help, and were reassured by mental health professionals that their sexuality was not a pathology and did not require ‘treatment’.

The following sections outline the examples we found of women receiving aversion therapy in England. These are drawn from a variety of sources. We found examples in published anthologies of British lesbian, gay, and bisexual lives. We also found examples in raw data from an unpublished survey of gay, lesbian, and bisexual people who had received psychiatric treatments in the UK.^4^ This survey was conducted in 1967 by the psychologist Eva Bene, in collaboration with the Albany Trust, a specialist counselling service for gay and bisexual people. The data was in the form of 237 completed questionnaires, including 44 from women. Whilst the survey questionnaire was poorly designed, making it difficult interpret the results, three women clearly reported receiving aversion therapy. We also found examples in published psychological research studies about aversion therapy treatment at a Crumpsall Hospital in North Manchester, where we also found press reports about an anonymous donation given in 1964 for a unit to expand existing research work into treating homosexuality.^5^ Finally, we found a reference to another example in a letter to a gay newspaper.

We supplement these examples with more detailed information from a woman who experienced aversion therapy in Manchester. She found out about our research and sent us her handwritten MA essay, where she had analysed her experiences.^6^ She gave us written permission to quote from this, and our subsequent conversation, which was used to clarify the information given. Names are used only where they were referred to in published documents, such as the first two examples, which we found in published LGBT anthologies.

## ‘Classic’ aversion therapy

Maureen was a teenager in the early 1960s, when she was subjected to the ‘classic’ type of behavioural-based therapy. It is not clear in which hospital the treatment took place, but as she was interviewed for Gardiner's research about the Gateways Club in London, one of the few lesbian venues in the UK at the time, it was probably in the London area. Maureen recalled:

The psychiatrist told my parents about me being a lesbian, and against my will, my mother signed a consent from for aversion therapy in the hospital. For the next six weeks I was given injections [to induce vomiting] and electric shocks when pictures of women came up on screen. I was made physically ill at the sight of women doing anything. For three months I felt terrible. It put me off women. I could not face being anywhere near them. What it didn't do was make me like men. (Gardiner, 2013: 62)

A remarkably similar example is given in an anthology about lesbian and gay lives in Brighton (Brighton OurStory Project, 1992). Janice was 20 years old when she had a mental health-related breakdown and was admitted to Graylingwell Hospital in Chichester, West Sussex in 1964. Whilst on the ward, she started an affair with another female patient and told her psychiatrist about it during one of their sessions. As it was assumed she was under the age of consent, the psychiatrist told Janice's parents, and they agreed she should be treated for her homosexuality (actually, there was no official age of consent for sexual relations between women at this time). As a result, she recalls being involuntarily subjected to aversion therapy:

The psychiatrists told my parents about me being lesbian and this resulted in me being forced, against my will, to have aversion treatment in the hospital, which to this day I will never forgive them for. It was appalling to have to go through something like that. The treatment went over six weeks and the idea is you are given injections and made to feel physically ill at the sight of women doing anything. For about three months I felt dreadful about it, I mean, I couldn't face being anywhere near the proximity of women. But what it doesn't do, you see, is make you like men any more.… But [the affair] didn't stop really, because all it did, once the treatment wore off, I’d learnt to be crafty. I no longer told the truth in these sessions. (ibid.: 35–6)

It seems necessary to comment on the strikingly similar ways that these two accounts are worded. This could be a result of shared experiences and the narrators having framed their narratives in response to similar cultural influences, most notably a broader LGBT+ community narrative about psychiatry. We also need to recognise the possibility that they may actually be the same woman (with different pseudonyms). However, given how the two women are described in the source material, this seems unlikely.

Three women who completed the Eva Bene survey had also experienced aversion therapy. One woman was recorded as being under 25 years old with a diagnosis of schizophrenia and had been a hospital inpatient more than once, for between three and six months, ‘because of psychological problems’. She reported having had aversion therapy with drugs administered by male psychiatrists to treat her sexuality. After the treatment, she said she was still sexually attracted ‘towards my own sex’, had sexual activity ‘always with my own sex’, and found sexual relationships ‘always emotionally satisfying’. Whilst she said she initially ‘had a real desire to overcome my homosexual tendencies because my homosexuality made me feel guilty and ashamed’, she made it clear that she had not really consented to the aversion therapy, but had wanted treatment for her other mental health conditions, some of which had been related to her feelings about her sexuality. In a note at the end of the survey, she wrote:

I was lesbian and had treatment for schizophrenia and lesbian tendencies. I got well from depression and feelings of guilt, but still remain lesbian and would not change it for anything. So for schizophrenic tendencies – these have cleared up with drugs but they tried to change my lesbian tendencies but I did not want to change and am happy this way.

Two other women in Bene's survey reported having ‘sought treatment’ for their sexuality and received electric shock-based aversion therapy. One was a ‘bisexual female aged 25–45’ who said she had sought treatment as she had felt it was ‘almost impossible to lead a stable and satisfying life as a homosexual’, and the other a ‘homosexual female aged less than 25’ who had wanted to rid herself of her homosexual tendencies. Both women reported the therapy as having been unsuccessful, as they were ‘still attracted to women’, but also reported it as having been either of ‘some help’ or of ‘great help’. As no other information was provided, it is hard to make sense of this apparent anomaly.

## Anticipatory avoidance therapy

We found references in the psychiatric literature to four women being subjected to anticipatory avoidance therapy at Crumpsall Hospital in North Manchester in the mid to late 1960s (Feldman and MacCulloch, 1964; MacCulloch and Feldman, 1967). This technique was a revision of the classic aversion therapy treatment, which was developed by behavioural psychologists Feldman and MacCulloch to treat male and female homosexuality (MacCulloch, Birtles, and Feldman, 1971). In this procedure, patients could ‘choose’ to not receive the electric shock if they pressed a button to remove the sexualised female image, within a certain time limit, and replace it with a male image (or vice versa, depending on whether they were treating men or women). It seems likely that the anonymous donation referred to earlier was used to fund the development of this technique. Unlike the examples of classic aversion therapy in the previous section, in which the treatment was given when the women were inpatients, this treatment was administered on an outpatient basis.

In *Homosexual Behaviour: Therapy and Assessment*, Feldman and MacCulloch reported two different research studies that both appeared to include two female patients treated with this technique (Feldman and MacCulloch, 1971). It is not entirely clear whether these are different women, as there is little specific information provided about two of the women. However, a renowned behavioural therapist clearly stated, approvingly, that four female patients were treated with this method by the two psychologists (Bancroft, 1969).

One of the studies included information about two of these four women who received aversion therapy at Crumpsall Hospital between July 1963 and August 1965 (Feldman and MacCulloch, 1971; MacCulloch and Feldman, 1967). They were both 18 years old and had been having a sexual relationship with each other, as well as relationships with men, one of whom persuaded them to get medical help. One of the women visited her GP explaining that she was disturbed by her sexuality and was told about the treatment being developed at Crumpsall. As a result, she was ‘given anticipatory avoidance therapy and advised to disassociate from her lesbian partner [and] made steady progress in becoming sexually averse to females, including her former partner’ (Feldman and MacCulloch, 1971: 209). As she appeared to have benefited from the treatment, she and her boyfriend (whom she subsequently married) encouraged her former female partner to have the treatment too. According to the researchers, both women ‘successfully completed treatment’ showing ‘very good improvement and neither displayed any homosexual fantasy, interest or practice’ (MacCulloch and Feldman, 1967: 596).

We found out more information about her former partner who also received the treatment, as she wrote about her experiences in her women's studies essay, 20 years later.^7^ In our subsequent conversations, it transpired that she was one of the patients referred to in this study, as she recognised herself in the patient description (Spandler and Carr, 2020). Back in 1965, she was very unhappy, very isolated, and increasingly unable to see a future for herself as a lesbian. Ironically, she had started university studying psychology, where she had learned about behaviourism. At the time, she felt the idea she might have a ‘behaviour disorder’ was preferable to having a ‘sexual perversion’, which was the other main psychological option available within which to see herself, rooted in moralistic or psychoanalytic notions. Her position reflected the way behaviourism was able to situate itself within the psy disciplines, as more enlightened, progressive, and effective than either psychoanalysis or criminalisation. As a result, she decided to ‘voluntarily’ seek the treatment herself, although she later noted that there was ‘nothing else on offer’. The psychologists were so keen to treat her that they offered her a temporary job as a receptionist in the hospital during the summer of 1965, when she was 18–19 years old. This enabled her to receive the treatment, informally, during her lunch break. She estimated that she received about 20 sessions in total. A male clinical psychologist administered the first few treatments, but in ‘de-briefing’ sessions afterwards, where she was encouraged to redirect her sexual feelings away from women and towards men, she told the psychologists that she did not like the male psychologist being there. As a result, the remaining sessions were administered by a female psychologist. Her unpublished essay eloquently described the anticipatory avoidance technique she was subjected to:

Within weeks I found myself sitting in a chair, looking at a blown-up picture of an unknown, semi-clad female and waiting to receive an electric shock. The electric shock itself was not severe, but still painful. Perhaps more unpleasant was the anxiety and fear of waiting for the shock, the anxiety and fear that they were systematically trying to link with the female form on the screen.… Their particular technique was a refinement of previous ones where the subject receives a shock each time the female form appeared. For me, three things could happen: I could receive an electric shock immediately the photograph appeared; I would receive a shock after 30 s; or there would be no shock at all. They had discovered that the technique created far more anxiety and fear than the original one [where the person would just get a shock when the picture appeared]. The final refinement was that by pressing a button I could even avoid the shock. I could replace the female photograph with one of a man. I could avoid the physical violence of the electricity and the emotional violence of the acute anxiety by choosing a man.^8^

This quote astutely highlights how this technique created the impression that the patient was in control of the procedure, by being able to switch the slides. However, the only way to avoid receiving the electric shocks was to select a male image and effectively choose heterosexuality. She sensed that the psychologists were more interested in opportunities to advance their treatments and their careers than in really helping her. For example, they did not encourage her to accept her sexuality, rather than try to change it. After the treatments, she took the psychologists’ advice and continued to have relationships with men, but with little success. Eventually, after discovering feminism, she decided to ‘come out’ and not force herself to be heterosexual. In her essay, she reframed the experience of aversion therapy as ‘anti-lesbian emotional and physical violence’.

## Unspecified aversion therapy

We found a final example in a letter published in a gay magazine (*Gay News*, London). The letter mentioned a woman who had been subjected to an unspecified form of aversion theory in a hospital in the north-east of England in 1973 (Llewellyn, 1974). The author highlighted the abuse and indignity of psychiatric treatments for homosexuality. They wrote that this ‘may result in patient suicides … as evidenced by the actions taken by a gay woman after taking a course of this “treatment” in the north east of England last Easter’. Unfortunately, no more information was given about this situation, or how the author of the letter knew about it. The letter strongly implied the woman had taken her own life, although it is possible it was a suicide attempt or other form of serious self-harm. In the rest of the letter, the author explained they were trying to contact gay people who had received aversion therapy and identify which hospitals and doctors were administering it. No other information was provided about this example, or the research, either in the letter or in subsequent issues of the magazine, and we were unable to find other references in the archives we consulted. Whilst we were not able to verify this example, research and anecdotal evidence confirms that aversion therapy did cause gay people considerable distress (see e.g. d’Silva, 1996).

## Discussion

Our research was not intended to help us find out exactly how many women were subjected to aversion therapy, but to document the experiences of women who did, as it is often assumed that it was administered only to gay men. We still do not know how many lesbian, gay, bisexual, and trans people were subjected to these treatments. We were unable to access any hospital records for Crumpsall Hospital (now North Manchester General Hospital), where we know several women were given this treatment. It is possible that other individuals were subjected to these treatments at this hospital but were not included in Feldman and McCulloch's published research. Moreover, the woman we spoke to who received aversion therapy there told us it was administered ‘off the record’ as part of the psychologists’ research (although she did not remember having been informed of this at the time). Therefore, the treatments may not have even been documented in hospital records. Whilst these examples were probably rare, this makes it difficult to find any more examples.

In addition, we were able to access examples of only those people whose experiences were recorded in the archives we consulted, at the time we consulted them. Only a tiny proportion of lesbian, gay, and bisexual people's experiences are recorded in the archives, which are constantly being updated and added to. Moreover, if the treatments were ever ‘successful’ in orientating people to heterosexuality, or driving their sexuality underground, their experiences would be even more unlikely to feature in the archives. It is possible that other treatments of female homosexuality were hidden by the notion of female ‘frigidity’, where women were subjected to various treatment procedures to enable them to have sex with men, often at the behest of their husbands and partners (Davison, 2020b). In addition, we do not know how many women (or men) may have been subjected to aversion treatments in other countries. According to Sansweet (1975), the ‘anticipatory avoidance’ technique developed by Feldman and MacCulloch was exported abroad, where it was ‘adopted and adapted widely, particularly in clinics and hospitals connected with universities in the United States’, including Harvard (Birk *et al.*, 1971; see also Larson, 1970). It is worth noting that aversion therapy continued to be practised in the US until the 1980s. As it included a larger ‘sample size’ than many single-case-study reports, Feldman and MacCulloch's research also influenced the use of aversion therapy to treat homosexuality in Australia. For example, McConaghy experimented with a version of this technique with well over 200 homosexuals in Sydney, including up to 10 women (Davison, 2020b).

Furthermore, it was often difficult to establish how far women were actively coerced into treatment. Whilst some men were given the ‘option’ of aversion therapy, instead of prison, this was clearly not a free choice. Whilst women did not face this particular ‘Hobson's choice’, the isolation, guilt, and fear many women felt about their sexuality makes the notion of consent problematic. Patient consent is often difficult to establish in psychiatry generally, as mental health treatments are often administered through informal pressure or active coercion. In addition, there were significant pressures on women in the post-war period to conform to the social norms of getting married and having children, and this may have motivated some women to seek help (Jennings, 2008). Whilst some women may have technically ‘volunteered’ for these treatments, they did not do so completely willingly. They usually did so under duress, or through ‘encouragement’ from others.

Moreover, there were few alternatives available for women to view their sexuality in alternative and more positive ways (Jennings, 2008). After all, this predated the emerging gay and women's liberation movements, which challenged the oppression of homosexuality and female sexuality, and contested its pathologisation and treatment (Spandler and Carr, 2021). At the same time, our research suggests these women were not simply victims of aversion therapy, but some were also able to exert some control over their lives, despite these treatments (see also Davison, 2020b). However constrained their agency was, and however much they may have been deceived into believing they were exercising ‘choice’, or had control or over the process, some women were able to find ways of resisting. For example, lying to psychiatrists that the treatment ‘worked’ in order to get discharged, ‘coming out’ later in life, discovering feminism, and writing about their experiences.

## Identifying the need for further research

Ideally, further research would recruit oral history participants, especially female patients and professionals who may have administered the treatments, or worked with those who did, and either supported or challenged these practices. This would add more detail to the picture we have drawn. It would be interesting to find stories of any people who may not have ‘come out’ (as lesbian, gay, or bisexual) and led a heterosexual (or asexual) life after the treatment. Whilst this would not endorse the treatments as ‘successful’, it would give us an insight into the diversity of experiences and other possible historical forces and dynamics involved. However, given the small numbers involved, the lack of official records of these treatments in medical records, and the likelihood that the professionals involved have retired, this would be an extremely difficult task.

However, it would be possible to conduct oral history research with ex-psychiatric patients and the wider LGBTQIA+ community (especially LGBTQIA+ psychiatric service users and survivors). In addition, further research could explore the medical, psychiatric, and psychological treatment of lesbian, gay, and bisexual people in other counties where treatments like aversion therapy may have been imported, or other treatments developed. In addition, it is important to record the experiences of bisexual, asexual, and other sexual minorities who may have been psychiatrised. Moreover, as we have made clear, whilst this research has focused on treating (women's) *sexual orientation*, to convert them to being heterosexual, additional research is needed to explore gender-non-conforming and trans people's experiences of normalising psychiatric treatments to attempt to treat their *gender orientation*.

Therefore, future research could explore the histories of other forms of reorientation treatment and therapies for homosexuality, and other minoritised sexualities and genders. For example, it is highly likely that some men and women would have received psychoanalytic treatment, or other psychotherapies, for their homosexual feelings or gender incongruence. Indeed, many psychoanalysts explicitly touted psychoanalysis as an effective treatment for sexual and gender deviations, including female and male homosexuality, transvestitism, and transsexuality (O’Connor and Ryan, 1993; Rosen, 1964, 1979). For example, Albert Ellis (US) and Clifford Allen (UK) published several papers documenting the effectiveness of psychotherapy with males and females with ‘homosexual problems’ (Allen, 1940, 1958; Ellis, 1956).

It is possible that both psychoanalysis and behaviourism viewed their treatment of sexual deviations as a good way to demonstrate the effectiveness of psychological approaches to mental pathology, which, at the time, were viewed as preferable to treating it as a criminal or moral wrongdoing. Moreover, in the context of fierce rivalry between psychoanalysis and behaviourism, the treatment of homosexuality offered an unfortunate ‘test case’ for the effectiveness of their respective therapies. Whilst we did find several mentions of attempted psychoanalytic treatments of female homosexuality in the literature, patient experiences are likely to be even harder to find and document than aversion therapy. For example, it would be difficult to find examples of psychoanalytic treatment, because sessions are often conducted over many years and in private practice and, by definition, rarely have clearly defined treatment outcomes, unlike behaviourism.

## Concluding thoughts

It seems that aversion therapy was an experimental treatment, rather than a mainstream service response to homosexuality, especially female homosexuality, in England. As a result, the number of women who received such treatments was likely to be very small, certainly smaller than the number of men. However, regardless of the small numbers involved, this history is still important to the history of psychiatry and psychology, to the women subjected to these treatments, and to the broader LGBTQIA+ movement (Jennings, 2008; King and Bartlett, 1999; Smith, Bartlett, and King, 2004). Service users from sexual and gender minorities have remained largely absent from published histories of psychiatry. Moreover, lesbian and gay histories have tended, up until recently, not to include mental health service users, perhaps due to a wish to distance themselves from historical associations with mental disorder and pathology (Carr, 2017, 2019).

Therefore, our study contributes to the histories of psychiatric treatments and to the particular history of marginalised and pathologised sexualities. Our research also complements the existing histories of gay men's experiences of aversion therapy (Dickinson, 2015). It also contributes to recent attempts to highlight lesbian activism in the UK, both inside and outside the mental health professions, to contest the psychiatric pathologisation of homosexuality (e.g. Hubbard, 2019; Hubbard and Griffiths, 2019; Spandler and Carr, 2021). As a result, it is part of a broader ‘hidden from history’ project (Duberman, Vicinus, and Chauncey, 1989), which aims to recover and preserve LGBTQIA+ history, women's history, and mental health service user/survivor history. This research is also a powerful reminder of how the medical and psychiatric profession can be used to enact or challenge societal prejudices.

Lest we think that this history is no longer relevant to current practice, it is worth bearing in mind some important lessons. For example, our research helps to illustrate the dangers of experimental treatments conducted by researchers and clinicians eager to prove their psychological theories or advance their own particular ‘brand’ of therapy and treatment. In the context of disciplinary and sub-disciplinary ‘turf wars’, research can be used to attempt to prove or disprove wider theories about human psychology in order to ‘settle scores’ with rivalrous disciplines. The current context includes increasing pressure on researchers and clinicians to publish research, especially ‘outcome’-focused research, and whilst the rivalry between behaviourism and psychoanalysis may have receded, there is still evidence of competition between other forms of therapy and treatments. In the last decade, this has focused on fierce debates about the rise and dominance of cognitive behavioural therapies, which actually emerged out of behaviour therapy (Marks, 2015).

Given the role of clinical ‘evidence’ in the emergence and dominance of therapeutic approaches, it is also worth questioning the privileging of evidence and effectiveness, and challenging what is considered ‘successful’ outcomes. This is especially important when treating socially undesirable traits and behaviours. For example, even if aversion therapy had been ‘successful’ in reorientating sexuality, this would not have made it ethical. In this context, it is worth noting that behavioural modification techniques are still commonly used to ‘treat’ psychosocial diversities such as autism (Rutherford, 2009).

This history also has implications for how sexual and gender minorities may feel about using mental health services today. Even though only small numbers of women may have been directly subjected to these treatments, a far larger number would have heard about them. For example, individual stories about aversion therapy and other forms of psychiatric treatment probably circulated in gay and lesbian bars and groups (Jennings, 2008). This may have resulted in fear and antagonism towards mental health professions and added to LGBT+ people's reluctance to engage with services (Alencar Albuquerque *et al.*, 2016). Moreover, mental health services are often still experienced as hetero- and gender-normative, especially by older lesbian, gay, bisexual, and trans people, even if staff are not actively homophobic or transphobic (Carr, 2010). It also needs to be pointed out that whilst the specific technique of aversion therapy may have all but disappeared, the broader practice of ‘conversion therapy’ for sexual and gender minorities is still legal and practised in many countries, including England (International Rehabilitation Council for Torture Victims, 2020).^9^

Finally, professionals who treated homosexuality were not only lauded at the time, but also remained in prominent positions in institutions around the world and continued to receive awards for their contributions to the discipline (King and Bartlett, 1999). Elsewhere we have argued for a Truth and Reconciliation approach to psychiatric harm (Spandler and McKeown, 2017). This process would start by acknowledging the mistakes of the past and involve carefully and truthfully documenting this history. This could help to prevent future wrongdoing and begin to heal the harm caused by the psychiatric and psychological mistreatment of minorities.

## Notes

1. *LGBTQIA+* is an umbrella term referring to people with marginalised sexualities and genders, including lesbian, gay, bisexual, transgender, queer, intersex, and asexual (or aromantic). We also use *LGBT* and other similar terms when referring to archives or other sources that use these different acronyms.

2. We did find a few isolated examples of women seeing psychiatrists for specifically gender-related issues (see e.g. Timms, 1969) but none who were subjected to aversion therapy. Some of these were probably gender-non-conforming women, and some could have been trans men or non-binary.

3. We explored the following LGBTQIA+ archives: the Lesbian Archive (Glasgow Women's Library), including the Lesbian Archive and Information Centre Collection (LAIC) and the Glasgow Women’s Library LGBTQ Collections; the Lesbian Information Service Archive (Lancashire Archives, Preston); the Hall-Carpenter LGBT Archives (LSE Women's Library, London); the Hall-Carpenter Oral History Archive (British Library); the London Metropolitan LGBTQ Archives (City of London Library, Guildhall); the Lesbian and Gay Foundation archives (Manchester City Library); Leeds Feminist Library North; and the Gale online database *LGBTQ History and Culture Since 1940* (parts 1 and 2). We consulted the following lesbian anthologies: *Investing Ourselves: Lesbian Life Stories* (Hall-Carpenter Archives Lesbian Oral History Group, 1989); *It’s Not Unusual: History of Lesbian and Gay Britain in the 20th Century* (Jivani, 1997); *Daring Hearts: Lesbian and Gay Lives of 50s and 60s Brighton* (Brighton OurStory Project, 1992); *Women Like Us* (Neild and Pearson, 1992); *Now You See Me: Lesbian Life Stories* (Traies, 2018); *The Lesbian Reader: An Amazon Quarterly Anthology* (Covina and Galana, 1975); *From the Closet to the Screen: Women at the Gateways Club, 1945–1985* (Gardiner, 2003); *We’re Here: Conversations With Lesbian Women* (Stewart-Park and Cassidy, 1977); *Lesbians Over 60 Speak for Themselves* (Kehoe, 1989); *The Lives of Older Lesbians: Sexuality, Identity & the Life Course* (Traies, 2016); and *Just Take Your Frock Off: A Lesbian Life* (Bell, 1999). We consulted the following oral history archives*:* the Hall-Carpenter Oral History Archive (British Library), which contains 121 interviews with LGBT people, covering the time from the 1930s to 1987; and the Mental Health Testimony Archive (British Library), which holds 50 life story video interviews with mental health service users.

4. Eva Bene, unpublished data from survey of psychiatric treatments for homosexuality, 1967, Hall-Carpenter Archive (LSE, London), HCA/ALBANY TRUST/10/5-9.

5. *Birmingham Post*, *Manchester Daily Telegraph*, *The Guardian*, *The Times* (25 November 1964); *The Scotsman* (26 November 1964).

6. Collier, P. (1986) ‘Themes of Violence in Anti-lesbian Treatment: A Personal Account’, MA essay.

7. Ibid.

8. Ibid.

9. There are current campaigns to outlaw conversion therapy for LGBTQ people.
